# Supplementary material for: Microglial senescence contributes to female-biased neuroinflammation in the aging mouse hippocampus: implications for Alzheimer’s disease
Source: bioRxiv. 2023 Mar 10:2023.03.07.531562. Preprint. [Version 1] doi: 10.1101/2023.03.07.531562 (PMC10028852; doi:10.1101/2023.03.07.531562)
Supplement: 1 [file NIHPP2023.03.07.531562V1-supplement-1.pdf]

bioRxiv preprint doi: https://doi.org/10.1101/2023.03.07.531562; this version posted March 10, 2023. The copyright holder for this preprint (which was not certified by peer review) is the author/funder. This article is a US Government work. It is not subject to copyright under 17 USC 105 and is also made available for use under a CC0 license.

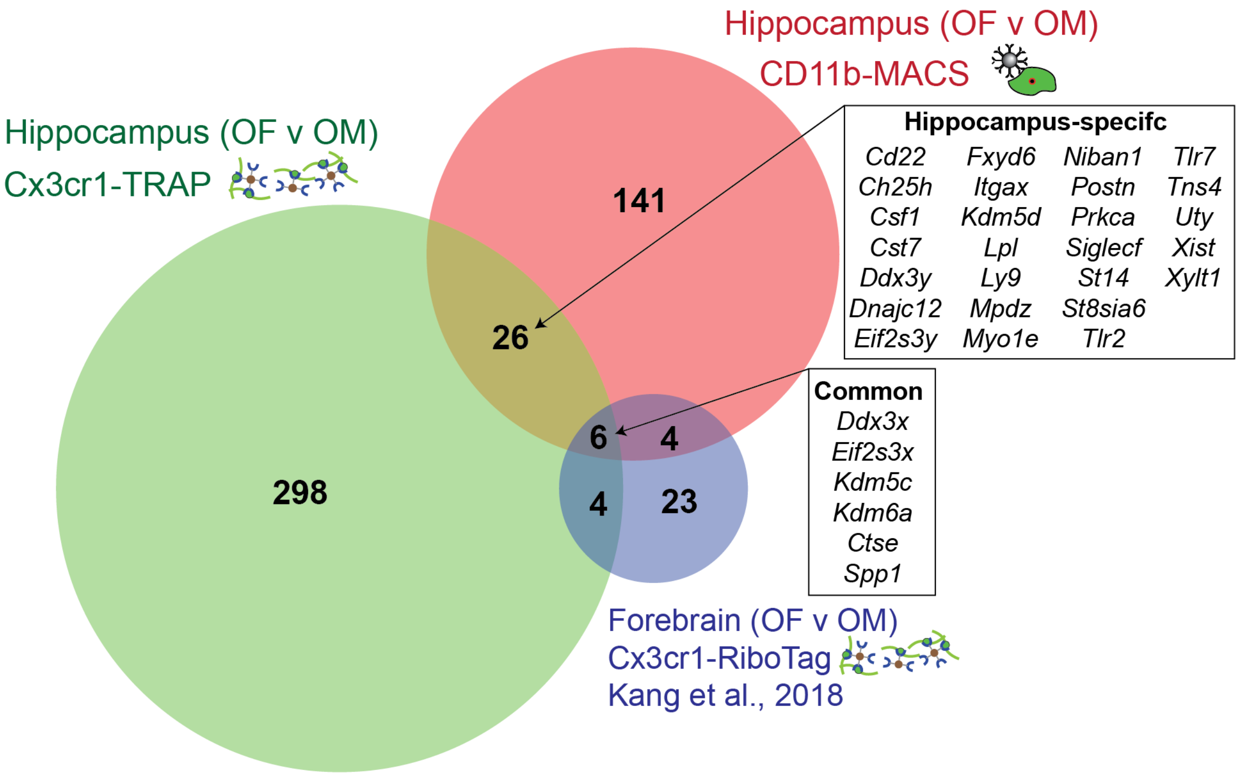

Supplemental Figure 2. Comparison of sex effects in aged microglia (22-25 mo) from the present study (hippocampus) and a previously published study (Kang et al., 2018; forebrain) identifies hippocampus-specific sex effects.
